# Role of bacteriophages in shaping gut microbial community

**DOI:** 10.1080/19490976.2024.2390720

**Published:** 2024-08-21

**Authors:** Md. Rayhan Mahmud, Sanjida Khanam Tamanna, Sharmin Akter, Lincon Mazumder, Sumona Akter, Md. Rakibul Hasan, Mrityunjoy Acharjee, Israt Zahan Esti, Md. Saidul Islam, Md. Maksudur Rahman Shihab, Md. Nahian, Rubaiya Gulshan, Sadia Naser, Anna Maria Pirttilä

**Affiliations:** a Department of Production Animal Medicine, Faculty of Veterinary Medicine, University of Helsinki, Helsinki, Finland; b Department of Microbiology, Jagannath University, Dhaka, Bangladesh; c Department of Biology, Texas A&M University, College Station, TX, USA; d Department of Microbiology, Stamford University Bangladesh, Dhaka, Bangladesh; e Department of Molecular Systems Biology, Faculty of Technology, University of Turku, Turku, Finland; f Department of Oncological Sciences, University of Utah School of Medicine, Salt Lake City, UT, USA; g Ecology and Genetics, Faculty of Science, University of Oulu, Oulu, Finland

**Keywords:** Bacteriophage, gut virobiota, gut microbiota modulation, prophage activation, phage therapy, antimicrobials, phage and human immune system, modulation of gut metabolites

## Abstract

Phages are the most diversified and dominant members of the gut virobiota. They play a crucial role in shaping the structure and function of the gut microbial community and consequently the health of humans and animals. Phages are found mainly in the mucus, from where they can translocate to the intestinal organs and act as a modulator of gut microbiota. Understanding the vital role of phages in regulating the composition of intestinal microbiota and influencing human and animal health is an emerging area of research. The relevance of phages in the gut ecosystem is supported by substantial evidence, but the importance of phages in shaping the gut microbiota remains unclear. Although information regarding general phage ecology and development has accumulated, detailed knowledge on phage-gut microbe and phage-human interactions is lacking, and the information on the effects of phage therapy in humans remains ambiguous. In this review, we systematically assess the existing data on the structure and ecology of phages in the human and animal gut environments, their development, possible interaction, and subsequent impact on the gut ecosystem dynamics. We discuss the potential mechanisms of prophage activation and the subsequent modulation of gut bacteria. We also review the link between phages and the immune system to collect evidence on the effect of phages on shaping the gut microbial composition. Our review will improve understanding on the influence of phages in regulating the gut microbiota and the immune system and facilitate the development of phage-based therapies for maintaining a healthy and balanced gut microbiota.

## 1. Introduction

Bacteria, viruses, fungi, multicellular parasites, and archaea constitute the human gut microbiota, forming an intricate and dynamic ecosystem with a density of 10^13^-10^14^ cells/g of fecal matter. This makes the gut microbiota the most complex ecosystem currently known. This complexity is due to significant variations in physical conditions and abiotic and biotic factors, including pH, oxygen, nutrient, and water availability, immunoregulators, and bile acids.^1^ Remarkably, every gram of human gut material is estimated to contain a minimum of 10^8^-10^9^ virus-like particles (VLPs).^2^ Frederick Twort (in 1915) and Felix d’Herelle (in 1917) initially described the existence of phages, bacteria-specific viruses that can kill bacteria.^3^ Unlike broad-spectrum antibiotics, phages typically exhibit high selectivity, targeting only the species or strain of host bacteria, making them a more refined approach for microbiota manipulation. Consequently, phages have garnered attention as potential regulators of gut ecology, as they not only influence bacterial populations but also impact the human immune system.^4^

The composition of the viral community in the gut exhibits a dynamic nature during the early life, characterized by a continuous turnover of phage species.^5^ The phages are believed to have initially hitchhiked to the gut system on pioneering bacteria as genome-integrated prophages, which are subsequently activated in the gut environment.^6^ Lysogens, which are bacteria carrying integrated prophage genomes, are abundant in the gut and may aid the survival of virulent coliphages in the infant’s gut.^7^ The colonization of phages is facilitated by the increasing diversity of bacterial species in the gut over time, as virulent phage families, such as Microviridae and crAss-like phages, are identified during later infancy.^5^ The human gut virome remains stable for up to one year. The persistent virome of adults is highly individualized, where a prevalent portion of viruses form a continual personal virome.^8^ The role of prophage activation in the stable adult gut remains undetermined. Recent evidence suggests that spatial heterogeneity within the gut, such as variations between the lumen and mucosal surface, may be a primary factor influencing the coexistence of phages.^9^ Similar to the human microbiota, the animal microbiota, such as those found in the cow rumen or pig digestive tract, exhibit a high diversity of phages.^10^ Phages significantly impact animals by shaping their gut microbiota, contributing to shifts from health to disease within the digestive tract ecosystem.^11^

The virome has emerged as a potential missing piece in the understanding of gut dysbiosis. Microbial dysbiosis in a healthy gut environment occurs when the homeostatic balance is disrupted. Typically, the healthy gut environment maintains a mutually beneficial interaction by providing metabolic and immunologic benefits to the human host. Once dysbiosis occurs, this mutual relationship turns to disruption, sometimes contributing to disease states. Dysbiosis in a healthy gut can be associated with overgrowth of the mucosal temperate phage populations.^12^ A temperate phage, sometimes referred to as a lysogenic phage, is one that amalgamates the genome into the host bacterial chromosome as a prophage.^13^ Distinct viral signatures have been identified in viromes associated with conditions such as colorectal cancer, type 2 diabetes, and inflammatory bowel disease (IBD).^14–16^ Inflammation, a hallmark of IBD, can activate the bacterial SOS system, leading to prophage induction and activation.^15^ However, the significance of the altered virome in disease development is not yet fully understood.

Phages have been studied extensively and tested in the treatment of various bacterial infections. Phages offer several advantages over conventional antibiotics in such treatments. Unlike antibiotics, phages possess a high degree of precision, selectively targeting the pathogens they recognize, which makes them more effective.^17^ Phages can replicate within the target bacterium without infecting mammalian cells or causing any side effects, resulting in improved safety and tolerability compared with antibiotics.^18^ Phages also have a longer duration of action, requiring fewer administrations over a shorter time, and can persist in the body for several days, or even longer.^19^

This review provides a comprehensive analysis of the role of phages in shaping the gut microbial community of both humans and animals, with a focus on composition, ecosystem, and impact on the microbial interactions with the animal or human host. We highlight the importance of phages in maintaining a healthy gut microbiota and activation of prophages in the gut. We also discuss the potential of phage therapy in combating multidrug-resistant bacterial pathogens and the possible immunomodulatory role of phages in altering the tumor microenvironment for anticancer effects. Finally, we discuss the influence of phages on the human immune system, elucidating their ability to induce innate and adaptive immune responses and their role in transferring virulence genes. By presenting these interconnected aspects, this review sheds light on the multifaceted impact of phages on the gut microbiota and their potential implications on human and animal health.

## 2. Composition and ecosystem of phages

### 2.1. Early development of the phageome

The colonization process of phages in the human intestinal tract shares a similar pattern to gut bacteria and begins during the first months of life.^20^ At birth, the neonatal gut is sterile from phages. During the first week of life, a fundamental phage colonization occurs principally through induced prophages of the evolving gut bacteria. As the phages colonize the human intestine, their composition changes over time. The diversity of the phageome (the entire community of phage populations) is initially minimal, and the sources of inoculation are unknown.^21^ The phages may be acquired from environmental sources, such as the birth canal,^22^ maternal gut microbiota,^23^ or breastfeeding.^24,25^

Several prophage-carrying *Bifidobacterium* species can be vertically transmitted from the mother’s breast milk to the newborn’s gut.^25^ As phage dynamics in the fetal gut are poorly understood, it is unknown how the *Bifidobacterium* prophages affect gut homeostasis.^5,20,26^ At the age of 15–24 weeks, *Caudovirales*, an order of viruses known as tailed phages, are dominant in the infant’s gut.^21^ The richness of *Caudovirales* phages decreases over the first 2 years of life,^27^ after which single-stranded DNA viruses belonging to the family *Microviridae* dominate in the gut.^21^ The inverse association between *Caudovirales* and *Microviridae* has been demonstrated by recent independent studies.^5^

In the following years, virulent phages of *Microviridae* and *Inoviridae* families become the most prevalent and shape the gut virome toward that of an adult.^5,28,29^ It has been hypothesized that in adulthood, the gut phageome is dominated by phages exhibiting a temperate lifestyle. The complexity of the full phageome remains incompletely understood, as the measures of the phageome are not absolute and the temperate phage diversity includes an abundance of lytic phages.^21^

### 2.2. Composition and distribution of phages in the gastrointestinal tract

The phage population in the human gut is characterized by a high level of diversity and exhibits variation in its viral structure. The phageome consists of either DNA or RNA, which can occur as either double-stranded or single-stranded structures.^30^ Phages are classified into four categories according to their genome type: double-stranded (ds)DNA, single-stranded (ss)DNA, double-stranded RNA (dsRNA), or single-stranded RNA (ssRNA).^31^ Considering the distribution of phages among individuals, Manrique et al.^28^ categorized them into three groups: (i) core phages, present in over half of all people, (ii) common phages, shared by many individuals, and (iii) low-overlap phages (or unique phages), found only in a limited number of people.^28^ Their study identified a set of 23 ‘core phages’ in more than 50% of healthy individuals from different geographic locations, 132 ‘common phages’ were found in 20–50% of the individuals, and as many as 1,679 ‘low-overlap phages’ were found in 2–19% of the individuals.^28^ According to a recent study conducted by Shkoporov et al. *CrAssphage* and *Microviridae* were identified as the most stable members of the gut viral population.^8^ This suggests that they may play a significant function as part of a core phageome.

The abundance of phages gradually rises throughout the gastrointestinal tract, progressing from the small intestine to the large intestine.^32^ Research suggests that the human gut has roughly 10^15^ bacteriophages,^33^ with an average of 10^8^-10^9^ bacteriophages per gram of human feces.^34^ The quantity of bacteriophages in the stomach rises significantly shortly after birth, with newborn feces containing 10^8^ phage particles per gram of feces at the age of 1 week.^35^ Interestingly, phage populations predominate in the intestines with eukaryotic viruses having a minor presence.^36^ Within a group of individuals that included both healthy and unwell people, it was shown that phages constituted the overwhelming majority (97.7%) of gut viral genomes. Eukaryotic viruses accounted for 2.1% of the genomes, while archaeal viruses made up just 0.1%.^2^ Remarkably, around 90% of the phage population was found to be unclassified, while the remaining phages identified belonged to the non-enveloped DNA phage group, specifically falling under the dsDNA order *Caudovirales* or the ssDNA families *Microviridae* and *Inoviridae*.^8^

Studies conducted on primates have shown that the presence and composition of phages in the gastrointestinal system vary depending on the specific location within the tract.^20,37^ Primates were used as animal models to collect samples from several sections of the gut, including the terminal ileum, proximal colon, distal colon, and rectum. Analysis of these samples revealed variations in the amount of phages across different regions. The relative abundance of *Microviridae*, *Myoviridae*, and *Siphoviridae* in the virome of the proximal colon was greater compared to that of the terminal ileum. Comparatively, the *Microviridae* and *Siphoviridae* were more abundant in the distal colon, whereas the rectum had a larger abundance of *Microviridae*, in contrast to the terminal ileum.^37^ Another comprehensive analysis of viruses concentrated on the gastrointestinal tract of two representative mammals, domestic pigs and rhesus macaques. The study was conducted utilizing metagenomics and confirmed the presence of variations in phage composition and abundance in different sections of the gastrointestinal tract. Both animals exhibited greater phage abundance and variety in the large intestine compared to the small intestine, however some viruses and phages were found in both the proximal and distal regions. The large intestine of pigs was mostly inhabited by the tailed phage *Caudoviricetes*, whereas the large intestine of rhesus macaques was primarily inhabited by *Microviridae*. The small intestine was colonized by a mixture of phages and eukaryotic viruses. Similar to other studies, the colon harbored the greatest amount and variety of phage biomass, mostly due to the high concentration of bacterial hosts in that specific region.^38^

Virulent phages have often been detected in the gastrointestinal tract of individuals suffering from intestinal disorders. These phages can originate from the activation of prophages in the gut bacteria during periods of stress. Prophage induction can cause intestinal dysbiosis by altering the balance between symbionts and pathobionts.^39^ Such disruption of microbial composition has been associated with various gastrointestinal disorders, such as *Clostridioides* (formerly *Clostridium*) *difficile* infections (CDI) and inflammatory bowel diseases (IBD).^40,41^ Notably, different diseases (i.e., CDI and norovirus associated diarrhea, or ulcerative colitis and Crohn’s disease) have been associated with specific gut phage compositions.^40,42^ For example, compared to healthy individuals, patients with CDI exhibit a higher presence of *Caudovirales* phages and a decrease in their diversity, richness, and evenness. Similarly, patients with norovirus-associated diarrhea experience a decrease in *Caudovirales* richness and diversity, as well as a decrease in their abundance.^42^ In addition, a study by Norman et al. found that patients with Crohn’s disease had a higher abundance of *Caudovirales* compared to healthy individuals. However, similar tendencies are not detected in patients with ulcerative colitis.^40^ These studies suggest a link existing between the presence of specific viruses and the general health of the gut.

## 3. Identification of novel human phages

The development of viral metagenomics has revolutionized our understanding of the human gut virome, leading to the discovery of numerous novel phage families.^43^ In 2014, a diverse, highly abundant phage family named CrAssphage was found by the culture-independent cross-assembly (crAss) method.^44^ *CrAssphages* exhibit a prolonged latent phase and a minimal burst size, allowing them to coexist with *Bacteroides intestinalis* in culture.^4^ Although their replication mechanism remains unassessed,^21^ they are believed to colonize the gut early in the childhood and to be vertically transmitted from mother to infant.^45^

Recent discoveries also include megaphages, or “Lak” phages, which have been discovered in the human fecal samples. They are characterized by their large genomes, consisting of up to 650 kbp.^46^ Megaphages are challenging to culture due to their high genome size, and thus molecular methods are required for their identification.^47^ Based on the CRISPR spacer targeting, a megaphage was predicted to replicate in the *Prevotella* genus, which is typically enriched in the gut microbiotas of people who consume non-Western diets in the developing world.^48^

Researchers have also successfully isolated and characterized a novel lytic phage named φPDS1, targeting *Parabacteroides distasonis*. Interestingly, φPDS1 belongs to a newly proposed genus and exhibits siphovirus morphology.^49^ Despite producing plaques, it lacks the genes associated with lysogeny and can coexist with its host in culture without significantly impacting bacterial abundance.^49^ This highlights the potential of phages in playing complex roles within the gut ecosystem, regulating bacterial populations without complete eradication.

Despite ongoing research, a substantial part of the human gut virome is yet to be fully understood. This uncharacterized fraction, often referred to as “dark matter,” remains largely unexplored due to several challenges.^50^ Even with the creation of databases containing over 50,000 viral operational taxonomic units (OTUs), most of these phages defy classification and lack assigned bacterial hosts.^51^ Several challenges impede progress in this field. Firstly, the absence of a universal phylogenetic marker gene shared by all phages hinders comprehensive identification. Secondly, sequence homology between these uncharacterized phages and currently classified phages within the International Committee on Taxonomy of Viruses (ICTV) taxonomy is often limited, further complicating classification efforts. Lastly, the lack of a widely accepted universal framework specifically designed for classifying novel and uncultured viral taxa creates additional hurdles.^52,53^ Furthermore, isolating novel gut phages in culture presents its own set of challenges. Difficulties can arise due to: (i) the inherent challenges of culturing specific gut bacteria; (ii) the limitations of traditional screening methods reliant on plaque or spot assays; (iii) the inability to recreate the specific environmental conditions within the gut necessary for phage replication; and (iv) the rapid emergence of resistance in bacterial hosts, for example, through mechanisms like phase variation.^54–56^ These limitations underscore the necessity for further research to develop innovative strategies for phage identification, classification, and isolation. Overcoming these issues will be crucial for unlocking the full potential of the human gut phage community and its role in health and disease.

## 4. Prophage activation in the gut

In general, bacteriophages are poorly characterized among the gut ecosystem. Specifically, there is a lack of information on their physiological significance compared to bacteria. Since 80% of the intestinal bacteria are thought to be lysogens, bacteria that contain prophages, the temperate phages are likely the most important in the gut.^57^ Recent studies indicate that the prophage activation (i.e. phage entry to the lytic cycle) has many implications in the intestinal environments. The prophage activation can affect adaptation and pathogen virulence of the bacterial host, composition of the gut bacterial community, and overall intestinal health.

### 4.1. Factors affecting prophage activation

The activation of prophages is primarily induced by several stimulators, including dietary factors, antibiotic use, certain bacterial metabolites, gastrointestinal transit, inflammatory environments, oxidative stress, and quorum sensing. The majority of phages are typically stable, but can be stimulated by external stressors or haphazard fluctuations in the phage repressor. The stimulation occurs when the prophages react to the host SOS signal and switch from lysogenic to the lytic mode, as described below. The phage repressor, which is usually an autocleavable protein dependent on host RecA, is the main element controlling this transition. In general, DNA damage and instability of the phage repressor are the biological cues responsible for prophage induction.^58,59^ The most widely discussed prophage inducers in the gastrointestinal tract are quinolone antibiotics, which result in DNA double-strand breaks.^58^ In particular, the use of quinolones to treat the shigatoxigenic *Escherichia coli* infection can induce a gastrointestinal disease and even the hemolytic-uremic syndrome, because of activation of the *E. coli* prophages that encode the Shiga toxin (stx).^60^ The role of prophage activation has also been studied in *Lactobacillus reuteri*, a gram-positive bacterium recognized as a gut symbiont of the gastrointestinal track in pigs, mice, rats, and birds. The study revealed that an acetate kinase (Ack) pathway is activated by exposure of bacteria to the short-chain fatty acids, acetic acid, propionic acid, and butyric acid, as well as hydrogen peroxide and bile acids, or combinations thereof, along with the metabolism of fructose. A fructose-enriched diet can accelerate the production of phages remarkably. Fructose can act as an electron acceptor when NADH is oxidized to NAD^+^, and fructose is converted to mannitol. Within the Ack pathway, the prophages are activated in a RecA-dependent manner.^61^

### 4.2. Mechanism of prophage activation

The two primary phage replication pathways are the lytic pathway and the lysogenic pathway. The lytic pathway activates genes such as holins and lysins, which cause cell lysis and lead to the production of new phages.^57^ On the other hand, the lysogenic pathway involves insertion of the phage DNA into the bacterial genome to produce a prophage that can be transferred to daughter cells. Some temperate phages may retain their external genes. The phage is kept in a lysogenic state by lysis repressors.^57^ The establishment of the lytic and lysogenic processes is influenced by environmental conditions and genetic predisposition. The lytic process permits quick reproduction and dissemination, but the lysogenic pathway forges a long-lasting bond with the bacterial host.^62^ In contrast to the lytic cycle, the lysogenic cycle does not result in generation of progeny virions following entry into the bacterial cell. However, the phages can produce DNA that is joined with the bacterial host chromosome.^63^ The combined part is called a prophage. The prophage can activate and enter the lytic cycle in response to the specific stimuli, as described above.

**Figure 1.**
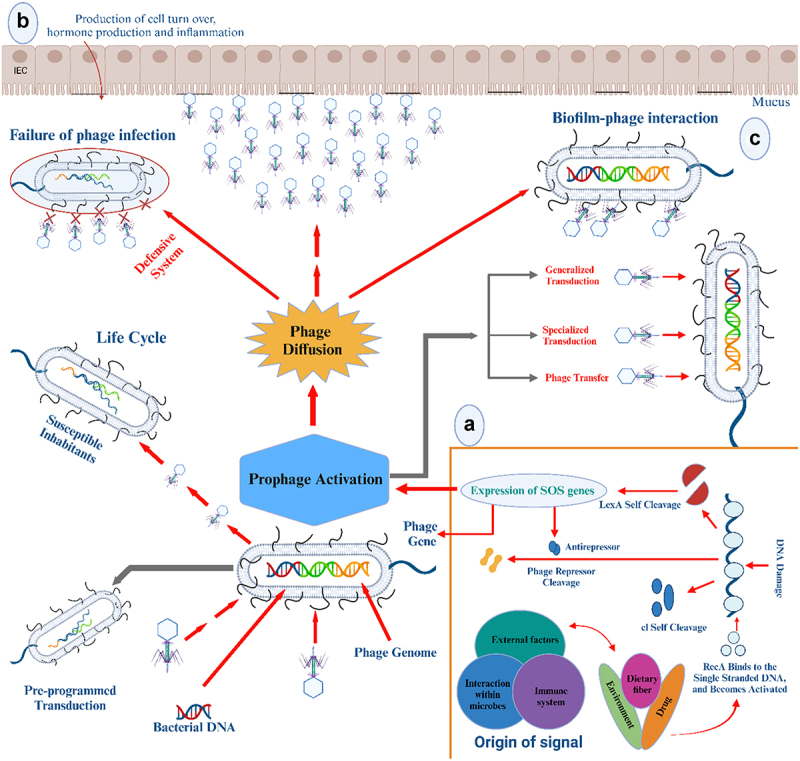
Prophage induction and diffusion of induced active phage. Several factors that can spontaneously trigger the prophage induction and diffuse the multiple cellular signals are presented. (a) The signal-triggering prophage activation, RecA protein, plays an important role in induction of the canonical pathway by binding to single-stranded DNA. Several signals, such as external factors from the environment or drugs, initiate the final expression of SOS genes after LexA and Cl act in self-cleavage. LexA or Cl-like phage repressors are then automatically cleaved by the nucleoprotein filament. The SOS genes are expressed when LexA repression is reduced, which starts the DNA repair and cell growth inhibition. Meanwhile, various genetic patterns are present in the lytic states and lysogeny. The master transcription repressor CI, which suppresses the lytic genes, maintains lysogeny. Upon DNA damage, the key sensor RecA is activated and leads to the CI self-cleavage, triggering the genetic switch to the lytic pattern. (b) Intestinal spread of the activated phage after induction of the SOS system in lysogenic bacteria. The process known as “auto-transduction” allows for phage release from a subpopulation of lysogenic bacteria to collect DNA from rival (from host) cells and transfer it to the remaining population. The two types of transduction—specialized transduction, in which neighboring bacterial DNA from prophages is excised and packaged into the capsid, and generalized transduction, in which random bacterial or plasmid DNA fragments are unintentionally packaged in the capsid—occur rather infrequently. The induced active phages can reproduce in a short lytic lifetime. (c) After the diffusion of the activated phage, it may attach itself to the bacterial cell to form communities in a biofilm environment. The mucus layer plays a more vital role in the phage enrichment than the surrounding tissues or cells. Illustration created in BioRender.com based on information from hu et al. Henrot and Petit 2022.^57,64^

The SOS response is mediated by two major antagonistic proteins that regulate the expression of SOS genes: RecA, an inducer, and LexA, a repressor. Prophages and the bacterial hosts have a commensal relationship, which is primarily sustained by inactivity of the SOS system. In general, the SOS system is a pathway that is responsible for bacterial DNA damage responses. Through the linking actions between the RecA and the LexA, the SOS system organizes cellular responses to DNA damage (Figure 1a). The LexA-occupied inactive promoter regions restrict expression of SOS regulon genes. The RecA protein generates an active RecA filament (known as activated RecA) on a single-stranded DNA in response to the DNA damage. This protein also functions as a coprotease, catalyzing the self-cleavage of LexA in a DNA-free form, most likely by lowering the pKa value of an important lysine.^57^

Production of active phages is induced by the SOS system activation (Figure 1a). In general, high levels of SOS gene expression are caused by reduced LexA levels, whereas the SOS system can be turned off by a reduction in the signal from RecA or LexA, and the canonical SOS response depends on LexA and RecA. The promoter of SOS genes contains an incomplete palindrome sequence called the SOS box (also known as LexA box), to which the LexA protein binds specifically and inhibits the production of the SOS gene. The epigenetic transition from lysogeny to the lytic state results in the cleavage of the CI master repressor by the RecA in the coliphage lambda. In the coliphage 186, LexA cleavage relieves the phage anti-repressor (Tum protein) inactivation. The Cro repressor, known as the lytic repressor that functions during the lytic growth, is also essential for the prophage induction (Figure 1a). However, there are alternative mechanisms that can trigger the prophage activation.^57^ Spontaneous prophage activation depends on the phage repressor cleavage or cell density. In a recent study, shiga toxin 2-encoding prophages were inducible in the deletion mutant *∆recA*, demonstrating that there are several different factors involved in the phage activation.^45^ Furthermore, acyl-homoserine lactones generated by *Pseudomonas aeruginosa* caused recA-deficient *E. coli* to form lambda phages in a co-culture system, providing clear evidence of SOS-independent prophage induction.^64^ The prophage activity can also be modified by specific counteraction of xenogeneic silencers, such as Histone-like Nucleoid Structuring (H-NS) proteins. For instance, in *P. aeruginosa* PAO1, a double depletion of the H-NS family proteins MvaT and MvaU activates the prophage Pf4.^65^ In *E. coli*, the suppression of the transcription termination factor Rho causes the induction of the lytic cycle.^66^

### 4.3. Spread of induced phages in the intestine

Temperate phages may display lysogenic conversion or transduction in certain conditions. There are two types of transductions. In the specialized transduction, the flanking bacterial DNA of prophages is excised and packaged into the capsid, and in the generalized transduction, random bacterial or plasmid DNA fragments are unintentionally packaged in the capsid, which both occur rather infrequently.^67^ Although all phages can transduce, the transduction rates differ greatly across phages. The prophage activation causes a lysogenic conversion, leading to the release of a large number of virions. The prophage induction may intensify the negative interaction between bacteria and phages, resulting in a profound impact on the evolution of bacterial anti-phage systems (Figure 1b). In fact, bacteria have developed the anti-phage strategies at every stage of the phage infection process, including adsorption and DNA injection inhibition, abortive infection, toxin-antitoxins, and CRISPR-Cas systems.^68^

Although several pieces of evidence indicate the significance of temperate phages in the adaptation and evolution of bacteria (via lysogenic conversion, transduction, or auto-transduction), in most circumstances, the prophage induction has detrimental effects on the bacterial host.^64^ When stochastic fluctuations or environmental stressors induce prophages, the lytic cycle and subsequent lysis of the lysogen may recur (Figure 1b). For example, a recent study revealed that the phage generation had a deleterious impact on survival of *L. reuteri* during food digestion.^61^ The prophage activation has also been linked to a decrease in *Faecalibacterium prausnitzii*, a significant commensal bacterium in the human gut, in IBD patients.^63^

## 5. The role of gut phages in the human-microbe interaction

Phages play crucial roles in the maintenance of homeostasis as the outcome of their dynamic interplay with bacteria and the human host. The phage infection impacts many crucial properties of bacteria, such as growth and metabolism, antibiotic resistance, competition, and the pathogenic role of bacteria in disease. Through the outcome of these interactions, the human tolerance of phages can be determined, and the analysis of benefits and detrimental effects of phages in the human-microbiota interactions will become more accessible.^69,70^

**Figure 2.**
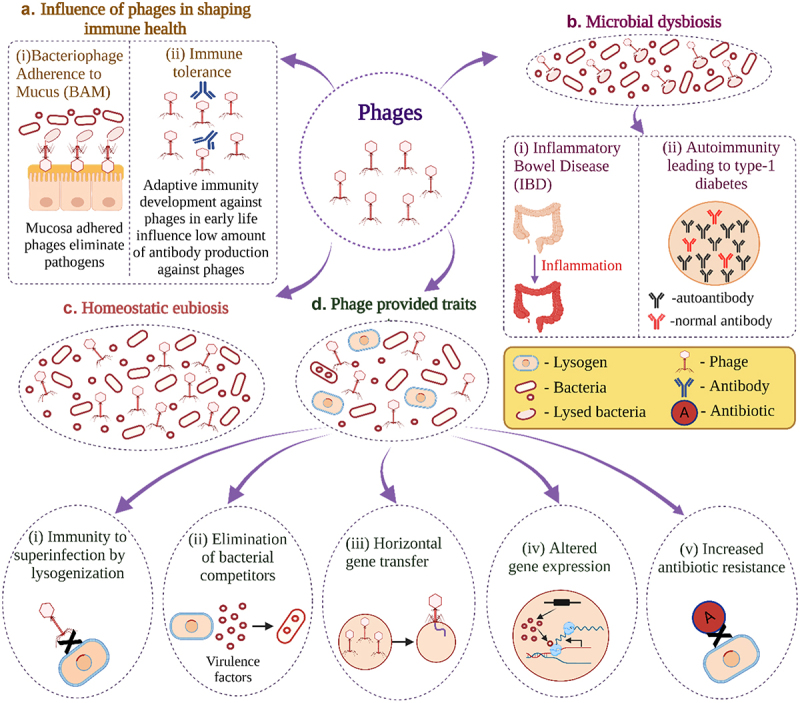
Role of phages in human-microbe interactions. (a) The influence of phages in shaping immune health; (i) bacteriophage adherence to mucus (BAM): pathogenic bacteria are killed by the mucosa-adhered phages; (ii) immune tolerance: human immunity tolerates phages by producing a low number of antibodies against phages due to the adaptive immunity development against phages in the early life. (b) Microbial dysbiosis due to phages can result in several diseases, such as (i) inflammatory bowel disease (IBD) and (ii) autoimmunity leading to type 1 diabetes. (c) Phages help maintain a homeostatic eubiosis inside the gut. (d) Phages provide several unique traits, for example: (i) lysogenization by phages provides immunity to the human host against superinfection. (ii) phages can provide virulence traits to pathogens when eliminating bacterial competitors; (iii) phages impart toxin-producing ability to microbes via horizontal gene transfer; (iv) phages alter normal gene expression; (v) lysogens (prophage-containing bacteria) possess increased antibiotic resistance. Illustration created with BioRender.com based on Chatterjee & Duerkop, (2018).^76^

Predatory lytic phages maintain the diversity of bacterial ecosystems. Absorbance and destruction of susceptible bacteria by lytic phages provide an immunological balance. The classic “kill-the-winner” model implies that virulent phages contribute to reducing the number of overgrowing bacterial species and act as a balancing factor to restrain niche monopoly by a single bacterial species. According to this scheme, phages do not attack bacteria that are present in low proportions despite the presence of an abundance of phages. Bacteria are lysed only when their overgrowth makes them prone to phage absorbance and predation.^71,72^ According to the “biological weapon” or “kill the relative” scheme, temperate phages prevail over non-temperate phages. In a natural environment, bacteria use their prophages as a biological weapon to eliminate bacteria inhabiting the same ecological niche. Although the sensitive population of the resident bacteria wanes at the early stage of prophage activation, lysogenization gradually makes the resident bacteria phage-resistant. As a result, prophages are not effective as a biological weapon for a prolonged period.^73–75^ In general, approximately 20% of the coding sequence in bacterial genomes are from temperate phages, and the host bacteria tolerate this high percentage of phage material in their chromosomes for several reasons (Figure 2d).^76,77^ Phages can provide immunity to superinfection by the lysogenization of the bacterial cells.^76^ They can also become internalized, releasing their nucleic acids inside eukaryotic cells. If these nucleic acids become degraded, they can trigger the immune system and contribute to interkingdom gene transfer.^74^

Dysbiosis in a healthy gut can be attributed to the overgrowth of the mucosal temperate phage populations by the “community shuffling” model.^12^ According to this model, the role of the temperate phages is negative on their host bacteria. In contrast to non-lysogens, a lytic phage kills its host bacterium if it senses even a mild stress.^78,79^ Numerous gut bacteria follow this pattern upon the use of subinhibitory concentrations of antibiotics.^79^ Inflammation can result from a deliberate lysis, and this phenomenon triggers a positive feedback loop. The outcome is a type of microbial dysbiosis (Figure 2b) that alters the relationship between resident commensal or mutualistic bacteria and pathogens.^78^ According to another, “Emerging New Bacterial Strain” model, the phages prefer establishing lysogeny by transferring genes instead of lysing the bacteria.^80–82^

It has been proposed that lytic phages may protect the human host from bacterial infections via an innate phage-mediated immune system.^83,84^ The phages attached to mucus have a reduced diffusion capacity, enabling them to efficiently eliminate the bacterial cells of low abundance. Significance of the lytic activity, and a high frequency of the lysogeny are further integrated. Thus, lytic activity may play a role in the lumen and other regions with low mucus concentrations in providing the human host a competitive advantage and acting as the first line of defense against bacterial invaders.^84^ By adhering to the mucin layer, phages protect the mucosal surface from pathogens. This is known as “Bacteriophage Adherence to Mucin” (BAM) [Figure 2a(i)]. Through BAM, the phages are enriched in the mucus by interacting with human mucin glycoproteins with their immunoglobulin-like protein present in the capsid.^83,85,86^ An antimicrobial layer is formed by the BAM, which prevents bacteria from attaching and colonizing the mucus layer and thus reduces epithelial cell death.^87^ Phages can also access the bloodstream by migrating through the epithelial layers.^86^

Overall, the role of phages in shaping the human-microbiota composition is poorly explored. Exposure to a diverse range of phages at birth can effectively influence immunological tolerance achieved in early life.^34^ The adaptive immune response against phages provides a weak immunogenic feedback. As a result, low titers of phage-neutralizing antibodies are produced (Figure 2a(ii)), which are insufficient to trigger an inflammatory response.^88,89^ Recent studies indicate that IBD is influenced by the increased level of double-stranded phage DNA (Figure 2b(i)),^47^ and IBD is associated with a transition of core virome from virulent to lysogenic life cycle.^29^ The abundance and diversification of phages during IBD are not disturbed by the gut bacterial community. Instead, phages transfer genetic material to pathogenic bacteria that provide environmental benefits (as stated in the “kill the relative” scheme earlier).^86^ Fluctuations in the intestinal composition of phages precedes the autoimmune development in type 1 diabetes (T1D) among children (Figure 2b(ii)).^90^ A study by Tetz et al. suggests that the amyloid-producing lysogenic *E. coli* can induce either seroconversion, or the development of type 1 diabetes in children. In-depth analysis suggests that the diabetes is caused by the *E. coli* prophages contributing to the bacterial amyloid secretion. When studied *in vitro*, the *E. coli* biofilm, an organized consortium of bacteria, released conspicuous amyloids when prophages were triggered by mitomycin C. Comparing this data to a metagenomic analysis, they discovered a similar phenomenon occurring in the gut of children who are developing either autoimmunity or T1D. The bacterial amyloid may induce islet amyloid polypeptide (IAPP) to pave the way for the breakdown of β-cell and β-antigen production, prominent in T1D. IAPP not only destroys β-cells but also triggers T1D by acting as an autoantigen.^91^

In the case of pathogenic bacteria, the phage-bacteria-human relationship is greatly affected by prophage-encoded toxins, or proteins that modulate antigens and effector proteins inside the human host. These toxins are not produced by the bacterial pathogen itself but rather in the presence of prophage-encoded genes. The bacterial pathogen must be lysed to release the toxin (e.g., botulinum toxin, stx). Pathogen virulence is the most well-known outcome of the lysogenic conversion.^75^ Lysogenic conversion by prophages produces various exotoxins and neurotoxins that are responsible for various common human diseases, such as cholera,^92^ scarlet fever,^93,94^ shigellosis,^95,96^ diphtheria,^97^ and botulism.^98^ In addition to the toxin production, antimicrobial tolerance of stx-encoding prophages of *E. coli* (STEC) is enhanced by the modification of bacterial metabolism. The lysogenic conversion transforms the bacteria from avirulent to virulent form due to the presence of stx-encoding prophages^76,99,100^ Polylysogeny, or the carriage of multiple prophages, is a common trait among pathogens that contributes to their diversity in disease pathology.^75,101,102^

## 6. Modulation of gut microbiota by phages

Through the above-mentioned mechanisms, phages can specifically target and infect the gut bacteria and eliminate them. Lytic phages, which kill bacteria, can also kill non-susceptible commensal species in the gut through cascading effects and continue their propagation through other microbial species to ultimately change the gut metabolome.^103^ However, phages do not always disrupt the microbiota, but may sometimes aid in the development of certain species. For example, administration of phages leads to a decrease in taxa associated with *Clostridium perfringens* and simultaneously increase in the number of *Eubacterium* species.^32^ Phages can also alter the pathogenic properties and biological functions of the gut microbiota during the lysogenic cycle. Besides the examples given earlier in the text, the activation of type III secretion system by the phage transcription factor Cro increases the virulence of *E. coli*.^82^ By providing genes that create resistance to antimicrobial substances and are involved in the metabolism of carbohydrates and polysaccharides, phages may play a crucial role in the proliferation of gut bacterial communities.^104^ Apart from directly affecting microbial species composition in the gut, the phages can influence human health through changes in the gut metabolites.

## 7. Phage-mediated modulation of gut metabolites

Gut metabolites, such as short-chain fatty acids (SCFAs), amino-acid derivatives, and bile salts, are small molecules produced by the gut microbiota that have a range of biological functions. They can act as signaling molecules that communicate with human cells, regulate immune function, and modulate metabolism.^105^ Alterations in the gut metabolite production are linked to various health conditions, such as obesity, diabetes, IBD,^106^ coronary artery disease,^107^ metabolic disorders,^108,109^ neurodegenerative disease,^110^ and cancer.^111^ The phage predation can modulate the production of gut metabolites, which play a significant role in the interaction between bacteria and their host, having implications for intestinal health. Specifically, the phage-induced changes in the metabolic products can influence host-microbiome crosstalk, potentially affecting immune regulation, inflammation, and metabolic homeostasis in the gut.^112^

In general, the phage-directed remodeling of the gut microbiota has a comparatively limited impact on the gut metabolome after a stable bacterial colonization.^103^ In a study, amino acids, peptides, carbohydrates, lipids, nucleotides, cofactors, vitamins, and xenobiotic metabolites were affected by the first set of phages by a significant percentage (17%). In contrast, the second set of phages affected only 0.7% of metabolites.^103^ However, a link has been discovered between the compounds produced and the specificity of phage predation on their target species. For example, in another study, the first set of phages enhanced the quantities of fecal serine and threonine, the two main amino acids of the O-glycosylated intestinal mucus, which is enriched by the mucin-degrading commensals *Akkermansia muciniphila* and *Bacteroides vulgatus*.^113^ Understanding the role of phages in modulating metabolic products in the gut is crucial for elucidating the complex interactions within the gut microbiome and their impact on intestinal health. Further research in this area may uncover novel therapeutic strategies targeting phages or their interactions with gut bacteria to promote human health or mitigate disease states. Next, we will list studies on specific groups of gut metabolites modified by phages.

### 7.1. Modulation of neurotransmitter metabolites

Metabolomic studies have revealed that phage predation in gut microbial communities results in lower neurotransmitter synthesis.^32^ For example, the neurotransmitter tryptamine, which comes primarily from plants but also from a small number of commensal gut bacteria, including *Ruminococcus gnavus* and *C. sporogenes*, was reduced following the treatment with phages.^114^ Another example concerns lactic acid bacteria, such as *E. faecalis*, which produces the neurotransmitter tyramine by decarboxylating tyrosine.^115^ Administration of the lytic phage VD13 decreased the number of *E. faecalis* and subsequently reduced the synthesis of tyramine.^112^

### 7.2. Impact on bile salt absorption and metabolism

Serum metabolites are suggested to correlate with phage presence and to be altered by them. For example, in one study, the bile salts were significantly altered by the first set of phages. Specifically, there was an increase in the deconjugated bile salts, such as cholate sulfate.^104^ In another study, a decrease in the conjugated salt taurochenodeoxycholic acid 7-sulfate was observed due to phage modulation of bacterial bile salt hydrolases. In general, the deconjugation and dehydrogenation of human tauro- and glyco-conjugated primary bile salts occurs by microbial bile salt hydrolases and hydroxysteroid dehydrogenases (HSDH), and the decrease resulted from increased bile salt hydrolase activity. There was also an increase of two specific bile salts, 12-dehydrocholate and ursocholate, associated with phage administration. The production of these bile salts resulted from deconjugation of secondary bile salts by 12α-HSDH, and sequential 7α-HSDH and 7β-HSDH activity.^103^ All these enzymes were associated with *B. fragilis*, *C. sporogenes*, and *E. coli*, which may decline by phage administration.^116^ On the other hand, the *Bacteroides* phage BV01 can suppress deconjugation of bile salts.^117^ The phages may also induce alterations in bile-salt absorption by the human host and in the bacterial ability to metabolize bile salts.^103^

### 7.3. Modulation of toxin and cytokine production

Several studies have reported the effect of phages on toxin production by gut microbiota. Besides the discussed cases of prophage-encoded toxins, such as stx and botulinum toxin, Wahida et al. observed reduced cytolysin production and inter-bacterial competition by phage-mediated *E. faecalis* removal. Cytolysin is a toxin produced by *E. faecalis* that causes cell injury and death to facilitate suppression of commensal *E. coli*.^118^ Phages can also induce the adaptive immune responses by activating B cells for antibody production and T cells for the production of cytokines, such as interferon γ (IFN-γ), through the Toll-like Receptor (TLR)-mediated signaling pathway (especially TLR9-dependent signaling pathway).^4,119^ Specifically, the phage predation affects cytokine production by the gut bacteria. Phages, such as phage 536_P1, may directly stimulate production of several antiviral cytokines, interferon, IL-12, and chemokines that may provide health benefits to the human host.^120^ A mouse model also revealed that oral introduction of T7 phages increased quantities of cytokines such as IL-1, IL-2, IL-12, and IL-17 in serum.^121^

## 8. The link between phages and the human immune system in the gut

Some studies have identified a link between phages and the human immune system in the gut, suggesting that phages can modify both innate and adaptive immune responses. Phage-mediated gene transfer and phage predation of bacterial species indirectly impact the immune responses and alter the human metabolism.^122^ Furthermore, the transfer of prophage virulence genes from the bacterial chromosome can be interpreted by the immune system as a potential pathogen, or true pathogens can evade the human immune surveillance due to prophage virulence gene transfer.^123^

**Figure 3.**
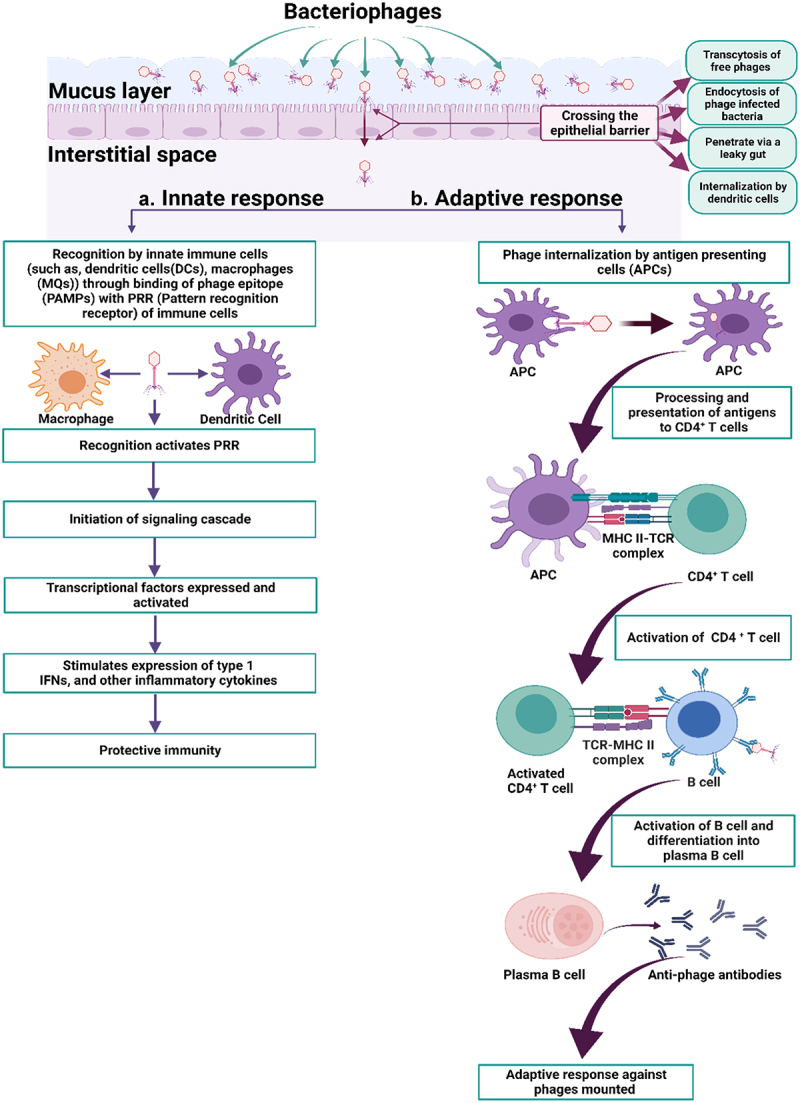
Link between phages and the human immune system in the gut. After crossing the epithelial barrier, phages can induce both the innate and adaptive responses. (a) **Innate response**: the phages are recognized by the innate immune cells, which subsequently stimulate a signaling cascade, producing type I interferon and other inflammatory cytokines, thus providing the human host with the innate protective immunity. (b) **Adaptive response**: the phages can also trigger a humoral immune response by inducing the production of anti-phage antibodies by plasma B cells. Figure is based on the cited references^4,120,123,126–128^ and created with BioRender. **PAMPs, pathogen-associated molecular patterns; PRR, pattern-recognition receptor; APC, antigen-presenting cell, MHC II, major histocompatibility complex class II; TCR, T-cell receptor; type I IFN, type I interferon.**

The principal function of the intestinal mucosal immune system is to maintain the intestinal homeostasis. This function is maintained by three immunological barriers, namely the mucus layer, epithelium layer, and immune cell layer.^124^ The mechanism of how phages influence the human immune response in the gut can be understood through the interaction of phages with the immune cells in the gut, followed by the induction of innate and adaptive immune responses.^4^ The interaction can be mediated indirectly by an association with the bacterial host,^4,125^ or directly, as the phages may interact with human immune cells by crossing the gut epithelium layer and eliciting an immune response.^126^ The overall process from crossing barriers to eliciting an immune response is summarized in Figure 3 and in the following section.^123,129^

### 8.1. Crossing the barrier

The phages are found in significant numbers in the intestinal mucosal layer, residing close to the epithelial layers.^123,128^ From there, the phages can penetrate the epithelial barrier mainly through a process known as transcytosis (or a phage-epithelial transcytosis).^128,130^ Other routes are through the endocytosis of free phages, through the endocytosis of phage-infected bacteria (the Trojan horse theory),^129^ by crossing the barrier when the tight cell-to-cell junctions are impaired (a condition known as leaky gut),^123,129^ through internalization of phages by immune cells (for example dendritic cells),^4,129^ and transcytosis via internalization of phages by immune cells.^123^

### 8.2. Recognition by innate immune cells

After crossing the gut-epithelial barrier, the phages are exposed to innate immune cells, such as dendritic cells and macrophages of the mucosal immune system.^129^ These innate cells possess specific receptors called pathogen recognition receptors (PRRs), such as Toll-like receptors (e.g., TLR3, TLR7, TLR8, TLR9), retinoic acid-inducible gene-I (RIG-1), and the cytoplasmic DNA sensor, cyclic GMP-AMP (cGAMP) synthase,^125,129^ which recognize the phage epitopes or the antigenic determinants called pathogen-associated molecular patterns (PAMPs).^125,126,129^ The following three primary mechanisms drive the recognition of phages by immune cells: 1) the cell-adhesion molecules i.e. the integrin or phage-specific receptor-driven extracellular recognition, 2) the endocytic recognition by endosomal PRR, and 3) the cytoplasmic phage nucleotide recognition through RIG-1-like receptors, or AIM2 (Absent in melanoma 2)-like receptors.^119,129,131^

### 8.3. Initiation of the signaling cascade for mounting an innate and adaptive immune response

The phage recognition activates the PRRs, which initiate a signaling pathway for viral-specific pro- or anti-inflammatory responses.^125,129^ The signaling cascade then activates the expression of transcriptional factors, such as NF-κB, IFN regulatory factor 3, and IFN regulatory factor 7. This transcriptional activation further stimulates the expression of type I IFN, IL-6, IL-1β, IL-8, and CXCL-10 to elicit an anti-viral immune response and continuous production of inflammatory cytokines.^125^

The phage recognition also induces a humoral response to provide durable immunity.^129^ The phage internalization by antigen-presenting cells activates the B cells to produce anti-phage antibodies. Opsonization of bacteria by phages (phage attachment to bacterial cell) contributes to more efficient phagocytic killing of bacterial cells, therefore providing protection to the human body against the bacterial pathogen.^128,132^

## 9. Influence of phages on human health

A key advantage of the use of phages as biological antibacterials, similar to antibacterial agents, such as antiseptics and antibiotics, is that they can be administered directly to the target tissue without causing injury or toxicity.^133^ The goal of the phage therapy is to use phages as a therapeutic agent to modulate the human microbiota to treat various chronic or degenerative diseases, such as dysbiosis in the gut or other niches of the body.^134^ The gut microbiota is associated with the intestinal health, and disruptions sometimes result in several chronic diseases.^135^ Living phages can be more effective therapeutic agents than conventional antibiotics. Therefore, using phages instead of antibiotics can promote intestinal health.^136^ However, researchers have varying opinions about the impact of phages on human health. There are investigations revealing that the phage therapy has a significant effect on human health, whereas other studies have shown negligible or a mild impact.^134,137,138^ For example, according to Tetz et al. 2017 administration of phages as supplements has only a low effect on the human body.^139^ Another, double-blind placebo-controlled study revealed that the phage uptake does not generally influence the gut microbiota, although the growth of specific gut members can be affected.^140,141^ In the study, a cocktail of *E. coli*-specific phages was given to healthy people for 28 days. Although an increase in *Eubacterium* and a decrease in the taxa *Clostridium* was observed, the phage did not completely eliminate any microbiota members.^140^ In these trials, the phage therapy was considered a safe and acceptable treatment for humans with some significant anti-allergic effects (a reduction in serum levels of the cytokine IL-4).^142^

Cieplak et al. also used the same phage cocktail in a small intestinal *in vitro* model to kill *E. coli*. This study suggested that compared to the antibiotic ciprofloxacin, the phage therapy has a more modest effect on the commensal non-targeted gut bacteria with stable phenotypic features.^143,144^ Both the phage cocktail and the ciprofloxacin have the ability to reduce *E. coli* with an average of 2.5 log CFU/ml (99.5%) reduction. The phage has a strong specificity toward *E. coli* and does not harm other members of the gut microbiota. In contrast, ciprofloxacin reduced other members of the community by an average of 1 log CFU/ml (90%).^143^ The study indicated that phages can be utilized in healthy individuals as a dietary supplement, although the phage supplement may cause mild gastrointestinal issues.^140^ However, the use of phages as anti-infectious agents also holds challenges which include the potential elicitation of an immune response due to their foreign antigenic load and genetic material.^138^ Another significant challenge is the development of bacterial resistance against phages which can occur through spontaneous mutations, making the bacteria resistant to phage attacks.^138^

Antibiotics significantly alter the bacterial community composition of the gut, as they not only eliminate harmful bacteria but also beneficial bacteria.^145^ Phages are a promising alternative to antibiotics and have the unique feature of inhibiting specific species or strains of bacteria without causing any harmful effects on other strains.^146,147^ Moreover, phages can be effective against bacterial biofilms, which can induce chronic infections due to their high resistance to antimicrobial substances, phagocytosis, and other components of the immune system.^148,149^ For example, the phage P100 exhibited a bactericidal impact on the biofilm surface when employed at 8 log PFU/cm^2^, being effective against 21 different strains of *Listeria monocytogenes*, a causative agent of food-borne infections.^150,151^ Hargreaves and Clokie et al. have proposed a phage therapy against CDI.^152,153^ In a recent study, the phage alone, or in a combination with vancomycin, reduced biofilms and prevented the colonization by *C. difficile*.^154^ Similarly, phages were successfully used to address a bloody diarrhea epidemic in Germany caused by stx-producing *E. coli*.^155^ Clinical and case studies that have been published on the use of phage therapy to treat human Gastrointestinal Diseases in recent years are shown in Table 1.

**Table 1.** Influence of bacteriophages on human gastrointestinal diseases.

Author and year	Participants	Infection type	Target Etiologic agents	Phage and dose	Duration of phage administration	Outcome and interpretation	Effect	Causes of Treatment Failure	Reference
Bruttin and BrüSsow (2005)	15 healthy adult volunteers	-	*Escherichia coli*	T4 phage; Two different doses (10^5^ PFU/ml & 10^3^ pfu/ml)	4 weeks	No differences were observed between the treatment and the placebo groups	Safe	N/A	156
Cepko et al. (2020)	Mice Model	Diarrhoea	*Escherichia coli*	Myoviridae phage PDX; (4.0×10^8^ pfu)	5 days	Inhibition of the target isolates and no effect of the normal human gut diversity in anaerobic culture were observed,	No adverse Effect	N/A	157
Corbellino et al. (2019)	57-year-old patient	Crohn’s disease	*Acinetobacter* *baumannii*	vB_KpnM_GF; (1× b 10^6^ pfu/ml)	3-week cycle	Elimination of the *Acinetobacter baumannii* was observed	No adverse Effects	N/A	158
Febvre et al. (2019)	43 healthy adults	Gastrointestinal issues	*Escherichia coli*	4 bacteriophage cocktails (LH01-Myoviridae, LL5-Siphoviridae, T4D-Myoviridae, and LL12-Myoviridae); 10^6^ phage titer per dose	28 days.	Reduction of *E. coli*, decreased pro-inflammatory bacteria, and increases in fermentative taxa were observed	Safe and tolerable	N/A	159
Federici et al. (2022)	Strains isolated from 18 BD patient - colonized in mouse model	IBD	*K. pneumoniae*	5 phages (10^9^ pfu/ml)	Three times per week (2 weeks)	Suppression of targeted strains and reduction of inflammation and disease severity were observed	Safe	N/A	160
Gindin et al. (2018)	32 healthy individuals	Gastrointestinal distress	*Escherichia coli*	4 supplemental bacteriophages Strains (LH01-Myoviridae, LL5-Siphoviridae, T4DMyoviridae, and LL12-Myoviridae); 15-mg capsule per day	28 days	No differences were observed between the treatment and the placebo groups	Safe and tolerable	lack of dietary assessment throughout the study may be seen as a weakness	141
Tetz et al. (2017)	5 healthy adult male Wistar rats	-	*Blautia, Catenibacterium, Lactobacillus*, and *Faecalibacterium* species	*Salmonella* bacteriophage cocktail and Pyobacteriophage; 1.5 mL, 1 × 10^6^ pfu/ml	10 days	Significant reduction was observed	No adverse Effect	N/A	161
Sarker et al. (2016)	120 children	Acute watery diarrhea	*Escherichia coli*	Microgen ColiProteus:1.4 × 10^9^ pfu, T4-like coliphage: 3.6 × 10^8^	4 days	Failed to improve the patient’s outcome and gut microbe’s amplification	Safe	Due to giving low phage titers dose and having insufficient phage coverage	162
Schooley et al. (2017)	68-year-old patient	Diabetic with necrotizing pancreatitis	*Acinetobacter baumannii*	9 phages in 3 cocktails (ΦPC, ΦIV, ΦIVB); (5×10^9^ pfu)	(ΦPC −18 weeks ΦIV −16 weeks ΦIVB −2 weeks)	Elimination of the target isolates, and patient returns to health	Safe	N/A	163
Shahin et al. (2020)	-	Acute gastrointestinal infections	44 *S. sonnei* and 26 *S. flexneri* isolates	Two bacteriophages (vB_SflS-ISF001 and vB_Ssos-ISF002); (10^9^ pfu/mL)	21 months	Inhibition of >85% of the *shigella* species were studied	No adverse Effect	Resistant pattern of the 5 isolates	164
Titécat et al. (2022)	Mice were colonized with 253 Strains	Crohn’s disease	*Escherichia coli*	EcoActive (7 phages); (2 × 10^4^ pfu/mL)	15 days	Reduction of targeted strains were observed in the gut of IBD patients	Safe	Single dose not efficient (1 × 10^9^ pfu/mL)	165
Vahedi et al. (2018)	48 mice		*Escherichia coli*	2 × 10^9^ pfu/mL	10 days	Completely reduction of the target isolates was observed	Safe	N/A	166

Note: ^a^Phage (PFU) or colony forming units (CFU) are listed if available in the original article.

## 10. Influence of phages on animal health

Equal to human health, we are beginning to realize the significance of phages in animal microbiota and health. Research on phage use in animals would also facilitate and expedite the clinical studies. However, further research is required to fully comprehend the wide range of effects and long-term implications of phage use in animal husbandry.^167^ In this section, we will list and discuss the numerous existing cases of phages found and used to treat animals.

### 10.1. Presence and role of phages in animal-microbe interaction

Besides the phages of bacteria, such as *E. coli, Salmonella, Bacteroides*, and *Klebsiella*, common in the feces of animals,^167,168^ several studies have reported phages in the rumen of sheep and cattle. The rumenous phages were associated with the bacterial hosts *Bifidobacterium ruminale, Streptococcus bovis*, *Streptococcus durans*, and *Prevotella bryantii*.^168,169^ Furthermore, the impact of phages on *Campylobacter jejuni* populations in the large intestine has been studied in chickens in more detail.^168,170^ *C. jejuni* is responsible of a large proportion of the bacterial food-borne illnesses globally, obtained through poultry. In a study, the presence of *C. jejuni* phages was inversely correlated with the level of gut bacterial colonization in the chickens, either as a consequence of lateral gene transfer, or the intragenomic inversions between Mu-like prophage elements.^168,170^

Phage resistance in *C. jejuni* is uncommon, because the strains that develop resistance are not efficient gut colonizers and easily revert back to the phage-sensitive genotypes. The phage resistance is developed through reversible genomic inversions, which lead to the activation of prophages in the genome.^171^ *C. jejuni* displays the genomic inversions when exposed to a virulent phage, and the *C. jejuni* cells can transfer genetic material to different strains through lateral gene transfer.^171^ For example, the genomes of strains R14 and R20 are specified through a recombination between the *CjE0227* to *CjE0241* genes, which subsequently produces the prophages R14-CampMu and *R*-20-CampMu, respectively.^171^ According to Scott et al. the strains R14 and R20 have three different phenotypes: 1) They produce the phage CampMu, 2) they have a poor colonization of the intestine, and 3) they become resistant to infection by the virulent phage CP34 that is unable to attach itself to the host bacteria.^171^ Due to these results, the phage therapy on *C. jejuni* is suggested as a highly potential method to control poultry contamination and prevent poultry-derived food-borne diseases.^171^

Despite these observations, little is known about whether phages impact innate and adaptive immunity in animals in natural interactions^74,122^ as in humans.^83,84,129^ In mouse tissues, the phage and its nucleic acids affect the expression of innate immune genes.^74,122^ However, in an experimental rat model, the phages alter the microbiota and increase intestinal permeability, suggesting that they may be detrimental to mammals.^139,172^ However, similar as in humans, the phages can reduce the microbiota imbalances in animals linked to disorders such as IBD, celiac disease, and metabolic syndrome.

### 10.2. Phage therapy in animals

Phage therapy has a longer history in animals than in humans. A summary of the experimental treatments and the effects of phages on animal health is shown in Table 2. For example, the disease caused by *P. plecoglossicida* was controlled by a phage in ayu, a Japanese fish.^173^ Matsuzaki et al. used a phage to protect mice against *S. aureus*, Cerveny et al. reported that a phage had a therapeutic benefit in the treatment of both localized and systemic murine *Vibrio vulnificus* infections, and Biswas et al. revealed that a phage could protect mice from lethal exposure to vancomycin-resistant *E. faecium*.^217–219^

**Table 2.** A summary of experimental treatments and effects of phages on animal health.

Participants	Etiologic agents	Bacteria titer^a^	Phage types	Phage delivery method	Phage titer^a^	Duration of phage administration	Key findings	Author	Reference
Fish	*Pseudomonas plecoglossicida* PTH-9802	10^7^ CFUs/ml	Mixture of PPpW-3 and PPpW-4	Oral	10^7^ PFUs/ml	2 weeks	The mortality of phage-treated fish was significantly lower than that of untreated fish.	(Park et al., 2000)	173
36-day old Broiler chicken	*Salmonella Enteritidis, Salmonella Typhimurium* and *S. hadar*	1 ml of 8.0 log10 CFUs/ml suspension of *S. Enteritidis*	151 phages against *S. Enteritidis*, 25 phages against *S. Hadar*, and 10 phages against *S. Typhimurium*	Oral	10^9-11^ PFUs/ml	24 hours	Phage reduced cecal colonization of *Salmonella enteritidis* and *Salmonella typhimurium* by 2-4 log units, respectively, within 24 hours	(Atterbury et al., 2007)	174
One-day-old chickens	*S. enteritidis*	2.4 × 10^5^ CFUs/ml or 7.9 × 10^5^ CFUs/ml	Phage cocktail (S2a, S9, S11)	Oral	10^6^ PFUs/bird	Different days and variables	10-fold bacterial reduction in chicken ileum, ceca, liver, and spleen	(Toro et al., 2005)	175
Leghorn chicken specific-pathogen-free (SPF) and mice	*S. typhimurium* ATCC 14,028ΩCm	Different doses	Three-phage cocktail (UAB_Phi20, UAB_Phi78, and UAB_Phi87)	Oral	10^10^ PFUs	Different days following	*Salmonella* concentration in chicken cecum decreased when the phage cocktail was administered 1 day before or immediately after bacterial infection and again on different days following infection. Result was not statistically significant in mice	(Bardina et al., 2012)	176
One-day old Broiler chicken	*S. enteritidis* PT4	100 µl at 10^8^ CFUs/bird	Three-phage cocktail (CNPSA1, CNPSA3 and CNPSA4)	Oral	10^11^ PFUs	Different days and variables	CFUs of *S. enteritidis* PT4 per gram of cecal content was reduced by 3.5 log units. A single dose with a high titer was more effective than a lower titer with long-term application.	(Fiorentin et al., 2005)	177
33-day-old Quails	*S. enteritidis*	1.2 × 10^9^ CFUs/ml	Siphovirus PSE (Single *Salmonella-*lysing phage)	Oral and vent lip	100 µl at 10^9^ PFUs/ml	Different times	Eliminated *S. enteritidis* from tonsils 6 hours after application. Phage PSE reduced *S. enteritidis* more effectively as a preventive agent than post challenge. Oral route is more effective than vent lip administration	(Ahmadi et al., 2016)	178
SPF chicks	*Salmonella sp*	Various doses	*Salmonella spp* phage	Oral	1.18 × 10^11^-1.03 × 10^2^ PFUs/chick	Several occasions	*Salmonella* loads were moderately reduced in cecal contents 3 days after infection (dpi) and significantly reduced at 5 dpi. From 7 dpi to the end of the study at 15 dpi, all chicks tested negative for *Salmonella*.	(Nabil et al., 2018)	179
Broiler chicks	*S. enteritidis*	Various doses	A mixture of phages (PHL 1-71)	Oral	2.5 × 10^9^-7.5 × 10^9^ PFUs	12 and 24 hours	*S. enteritidis* retrieved from cecal contents decreased at 12 and 24 hours after treatment compared to untreated controls.	(Higgins et al., 2007)	180
One-day-old chicks	*S. enteritidis*	5 × 10^8^ CFUs/ml	Phage CJ07	Oral	10^5^, 10^7^, and 10^9^ PFUs/g	21 days	Highest doses of phages significantly reduced pathogens from the digestive tract in the challenged and contact chickens. No intestinal *Salmonella* was found in 70% of contact chickens treated with 10^9^ PFU/g of phage.	(Lim et al., 2012)	181
7-day old chicken	*S. enteritidis*	2.95 × 10^5^ CFUs/ml	Three different *Salmonella*-specific phages and competitive exclusion	Aerosol spray	10^8^ PFUs/ml	7 days	The mixture of phages (BP) reduced *S. enteritidis* by 80%, and competitive exclusion (CE) reduced *S. enteritidis* to 75.7%. Combined CE plus BP reduced *S. enteritidis* to 38.7%.	(Borie et al., 2009)	182
6-week-old chickens	*S. gallinarum*	5 × 10^8^ CFUs/ml	*S. gallinarum*-specific phage	Oral	10^6^ PFUs/kg	7,14 and 21 days	Contacted hens with infected individuals receiving treatment with phage showed a considerable reduction in mortality (5%) compared with untreated group (30%).	(Lim et al., 2011)	183
One-day-old chickens	*S. enteritidis*	0.25 ml at 9 × 10^3^ CFUs/chick	Cocktails of different phages	Oral	10^8^ PFUs/chick	24-48 hours	Only effective for a short time with no long-term protection.	(Andreatti Filho et al., 2007)	184
Broiler chicks	*S. enteritidis*	10^5^ CFUs/g feed	*S. enteritidis* phage	Oral	10^8^ PFUs	14 days	Phage treatments on day 14 of the experiment considerably reduced prevalence of *S. enteritidis* in cloacal swabs.	(Kimminau et al., 2020)	185
Broiler chicks	*S. enteritidis*	Different titers	P22hc-2, cPII and cI-7 and Felix 0	Oral	5 × 10^11^ PFUs	14 days	Average cecal bacterial counts in phage-treated hens were 0.3-1.3 orders of magnitude lower than in control animals.	(Sklar & Joerger, 2001)	186
10-day old chickens	*S. enteritidis*	9.6 × 10^5^ CFUs/ml	Three lytic phages	Spray and oral	10^3^ PFUs	20 days	Prevalence of *S. enteritidis* infection was reduced by 72.7% after aerosol spraying of phages. Counts of *S. enteritidis* showed that drinking water and administering phages through coarse spray reduced intestinal bacterial colonization.	(Borie et al., 2008)	187
White leghorn chicks	*S. typhimurium*	~10^10^ CFUs/ml	Lytic phage (Φ st1)	Intracloacal inoculation	10^12^ PFUs/ml	Different times	*Salmonella* count decreased to 2.9 log10 CFU/ml within 6 hours after challenge. *S. typhimurium* was undetectable at and after 24 hours.	(Wong et al., 2014)	188
4-week-old weaned pigs	*S. typhimurium* ATCC14028	10^8^ CFUs/ml	Phage cocktail C (SEP-1, SGP-1, STP-1, SS3eP-1, STP-2, SChP-1, SAP-1, SAP-2)	Oral	≥10^9^ PFUs/ml	0-35 days (3 days interval)	Lytic activity against *Salmonella* reference strains was observed, Reduced *Salmonella* shedding in pig feces. Reduced number of species of Enterobacteriaceae family without disturbing the normal fecal flora	(Seo et al., 2018)	189
*Galleria mellonella* larvae and SPF mice	*S. aureus*	10^4^ CFUs/10 µl in larvae and 10^5^ CFUs/50 µl in mice	3 lytic phages (Romulus, Remus, and ISP)	Intra-hemolymph injection in larvae and intra-mammary injection in mice	10^7^ PFUs/10 µl in larvae and 10^8^ PFUs/50 µl mice	4 days post-inoculation in larvae and 48 hours post-inoculation in mice	Below 50% survival rate of larvae and incomplete recovery of the mice treated with IS phages in vivo.	(Ngassam-Tchamba et al., 2020)	190
Mice	Drug-resistant *S. aureus*	1 × 10^8^ CFUs/ml	Phage 4086-1	Intra- mammary injection	(1 × 10^8^ PFUs/ml)	NA	Proliferation of *S. aureus* in the mammary glands was significantly inhibited, and the TNF-α and IL-6 concentrations decreased after phage treatment.	(Teng et al., 2022)	191
3- and 7-day-old birds	*E. coli*	10^4^ CFUs/ml	Phages SPR02 and DAF6	Injection into the thoracic air sac	10^8^-10^3^ PFUs to air sac and 10^3^ or 10^4^ PFUs/ml in drinking water	Different times	Titer-dependent reduction in mortality rates.	(W. Huff et al., 2002)	192
Birds (1 week)	*E. coli*	6 × 10^4^ CFUs/ml	Two bacteriophage lytic to E.coli	Aerosol spray	10^8^ PFUs/ml and 10^4^ PFUs/ml	0-48 hours	Titer-dependent 35% reduction in morbidity rate and 100% reduction in mortality rate.	(G. R. Huff et al., 2009)	193
7-day-old birds	*E. coli*	10^4^ CFUs/ml	2 different phages DAF6 and SPR02 with enrofloxacin	Intramuscular	10^9^ PFUs/ml	7 days	Significant reduction of mortality rate to 15% in comparison with untreated groups. Combination of phage and enrofloxacin has a synergistic effect.	(W. E. Huff et al., 2004)	194
10-week-old chickens	*E. coli* (APEC H839E)	0.2 ml at 5.0 × 10^8^ CFUs/ml	Phage cocktail (phi F78E, phi F258E and phi – F61E)	Aerosol spray and drinking water	10^7^ and 10^9^ PFUs/ml	7 days	Reduction in mortality (25%) and morbidity (43%) by 10^9^ PFUs/ml phi F78E in experimental rooms. Low titer (10^7^ PFUs/ml) showed remarkable efficiency at large scale.	(Oliveira et al., 2010)	195
3-week-old chickens	*E. coli*	Different study counts	Phage SPR02	Sprayed	8 × 10^8^ PFUs/ml	7 days	Significant reduction in mortality by spraying on the litter. Reduced shedding of *E. coli* was among poultry flocks.	(El-Gohary et al., 2014)	196
10-day-old chickens, 32-day-old chickens	*C. jejuni*	1 × 10^5^ CFUs/g	Phages 71 and 69	Oral	10^9^-10^10^ PFUs	Different days	Infection inhibited by 1-2 log units in broiler ceca. Delay bacterial colonization if phages were introduced before infection.	(Wagenaar et al., 2005)	197
25-day-old chickens	*C. jejuni* HPC5	log10 2.7-7.8 CFUs/g	Phage cocktail (CP34 or CP8)	Oral	10^7^ and 10^9^ PFUs	5 days	0.5 to 5 log units of short-lived reduction of bacteria in the intestine.	(Loc Carrillo et al., 2005)	198
Chickens	*C. jejuni* HPC5 or *C. coli* OR12	8 log10 CFUs/ml	Phage CP220	Oral	5, 7, 9 log PFUs	Different days	Significant decrease in *C. jejuni* and *C. coli* with a density of 10^9^ PFUs.	(El-Shibiny et al., 2009)	199
31-day-old chickens	*C. jejuni* 2140 CD1	2.2, 1.1, and 5.8 × 10^6^ CFUs/g	Phage cocktail (phi CcoIBB35, phi CcoIBB37, phi CcoIBB12)	Feed and oral	10^6^ PFUs oral dose and 10^7^ PFUs in feed	2,4 and 7 days	Reduction in titer of both *C coli* and *C. jejuni* by approximately 2 log units. Reduction in incidence of campylobacteriosis by 30-fold.	(Carvalho et al., 2010)	200
One-day-old chickens	*C. perfringens* CP-6	10^8^ CFUs/ml at 1.0 ml/bird	Phage cocktail (CPAS-7, CPAS-12, CPAS15, CPAS-16, CPLV-42)	Feed, water, oral, and spray	10^5^ PFUs/ml	NA	Significantly reduced mortality rate after *C. jejuni* challenge. Significantly better ratios of weight gain and feed conversion.	(Miller et al., 2010)	201
3-week-old pigs	*S. Typhimurium*	10^3^, 10^8^, and 5 × 10^8^ CFUs	Salmonella-specific lytic phage (Felix-O1)	Oral and intramuscular	2 × 10^10^ PFUs/ml	3 and 9 hours	100% reduction of pathogens from tonsils after 6 hours	(Lee & Harris, 2001)	202
Lactating Cows	*S. aureus*	-	Single mixture (phage K)	Intramammary infusions	1.25 × 10^11^ PFUs/ml	5 days	No significant reduction in udder.	(Gill, Pacan, et al., 2006; Gill, Sabour, et al., 2006)	203,204
Steers	*E. coli* O157:H7 (ATCC 43,894, WSU180, WSU400, 588)	10^10^ CFUs	Single and phage mixture (SH1 and KH1)	Rectal and drinking	25 ml 10^10^ PFUs/ml via rectum and 1.8-5.4 × 10^6^ PFUs/ml	Different times	Bacterial reduction in the gut of steers was up to 1.5 log compared with control.	(Sheng et al., 2006)	205
Sheep	*E. coli* O157:H7 ATCC 43,894	3.5 × 10^10^ CFUs	Phage KH1	Oral and rectal	1.3 × 10^11^ PFUs	Various times	No reduction of bacterial load in the intestine	(Sheng et al., 2006)	205
Romanov weather lambs	*E. coli* O157:H7 strain E318N	10^8^ CFUs	Phage DC22	Oral	10^13^ PFUs/ml	Various times	Reduction in the first 13 days after inoculation except for one lamb. No changes at 30 days.	(Sheng et al., 2006)	205
Young feedlot steers	Nalidixic acid-resistant *E. coli* O157:H7	5 × 10^10^ CFUs	Phage mixture	Rectal and oral	Different titers	Different day counts	No significant reduction.	(Rozema et al., 2009)	206
Swine	*S. typhimurium*	2 × 10^10^ CFUs	Phage cocktail	Oral	3 × 10^9^ PFUs	24 and 48 hours	Reduction of *S. typhimurium* and other Salmonella strains from the intestine and fecal samples.	(Callaway et al., 2011)	207
Lactating Cows	*E. coli* and *Arcanobacterium pyogenes*	NA	Four-phage mixture	Intrauterine	10^7^ PFUs	Different times	No reduction in uterine isolation rate. No effect on reproductive performance.	(Machado et al., 2012)	208
Growing pigs (barrows)	*Salmonella (typhimurium, enteritidis, derby), S. aureus, E. coli* (k88, k99 and f41), and *C. perfringens* type A	NA	Phage cocktail	Feed	10^9^ PFUs/kg	35 days	Improved performance of growing pigs. Decreased CFU count of *S. enteritidis* by 3.5 log units.	(Kim et al., 2014)	209
Weaned pigs	*S. typhimurium*	5 × 10^8^ CFUs/ml	Phages specific to *S. typhimurium* with probiotic *L. plantarum* CJLP56	Oral	3 × 10^9^ PFUs/kg	Different times	Significant influence on growth and comparable protective effect with antibiotic-supplemented diet.	(Gebru et al., 2010)	210
3- to 4-week-old small pigs	*S. typhimurium*	5 × 10^8^ CFUs/ml	Phage cocktail	Oral	~10^9^ PFUs/ml	6 hours	90.0% to 99.9% bacterial reduction in the ileum, tonsils, and cecum samples. Influence on growth and weight of pigs.	(Wall et al., 2010)	211
Lactating cows	*S. aureus*	NA	Phage cocktail (K, CS1, DW2)	Infusions into teats	10^8^ PFUs/ml	0-8 hours	10 000-fold reduction of pathogen count.	(O’Flaherty et al., 2005)	212
Lactating cows with metritis	*E. coli*	NA	10 different phages	Intravaginal inoculation	10^9^ PFUs/ml	NA	No prophylactic effect observed. Cellular immune response parameters reduced.	(Meira et al., 2013)	213
Sheep	*E. coli* O157:H7 933	1 × 10^10^ or 2 × 10^10^ CFUs/sheep	Phage mixture	Oral	10^7^ or 10^8^ PFUs/ml	48-96 hours	Pathogen reduction in feces, cecum, and rectum.	(Callaway et al., 2008)	214
Sheep, crossbred ewes	*E. coli* O157:H7 EDL 933	10^10^ CFUs per sheep	Phage CEV1	Oral	~10^11^ PFUs	2 days	*E. coli* reduced in cecum and rectum. Prior phage treatment could delay bacterial colonization.	(Raya et al., 2006)	215
Cattle	5-strain mixture of nalidixic acid-resistant *E. coli* O157:H7	10^11^ CFUs/ml	Phage product with E phage with es rV5, wV7, wV8 and wV11 phages	Oral bolus gavage or feed	10^10^ and 10^11^ PFUs/ml	NA	No reduction with E phage. Reduction of *E. coli* in fecal samples by other phages.	(Stanford et al., 2010)	216
Iron-dextran-treated mice	*V. vulnificus* MO6/24-0	1150 CFUs	Phage 153A-5	Intravenous injection	10^4^, 10^6^, or 10^8^ PFUs	8 days	Only the highest phage dose provided protection.	(Cerveny et al., 2002)	217

Note: ^a^Phage (PFU) or colony forming units (CFU) are listed if available in the original article.

In general, the phages have been tested and successfully used in poultry in the treatment of various diseases. For example, *C. perfringens* causes necrotic enteritis, which can be prevented by INT-401 phages along with other endolysin-encoded *C. perfringens* phages. *S. aureus*,which is a problematic pathogen in chickens and turkeys,^220,221^ and is prone to resistance against antibiotics.^222,223^ A recent study proposed that a three-phage cocktail against *S. aureus* tested in mouse and *Galleria mellonella* models can be used in cattle.^190^ From a therapeutic standpoint, Myoviruses are the most promising staphylococcal phages.^224^ Bacterial phages from the Caudovirales order and the Siphoviridae family have also been tested and have high lytic properties against the *Staphylococcus* strains. However, these phages were ineffective for phage therapy due to enterotoxigenic genes.^225^ Furthermore, Xie et al. demonstrated that the phage Esc-A, originally obtained from sewage, was more effective than the antibiotic chloromycetin in reducing diarrhea and death rates in chickens.^226^ Previous research has also shown that phages may significantly decrease *Salmonella* spp. counts in internal organs of chicken, feces, and poultry products.^227^ Recently, *S. enteritidis* strains were shown to be less abundant when a single oral cocktail of phages was used.^228^

In addition to reducing the disease burden, the phage supplementation can enhance feed efficiency and liver weight in chickens and increase egg production and egg quality in laying hens.^228^ According to Wang et al. adding 0.5 g/kg of phage to the diet increased the chicken liver weight and feed efficiency.^227^ The phages affect the populations of gut bacteria, which are essential for the development of the liver and gut immune systems, but the exact mechanism of affecting chicken liver weight remains unclear.^227^ However, a recent study revealed that the bacterial host cell lysis by the phages can release enzymes, including those involved in carbohydrate fermentation.^229^ These enzymes can aid feed breakdown, improve ruminal fermentation, and provide energy for maintenance, growth, and production.^229,230^

Although the phages can reduce the burden of *E. coli* O157:H7 in the intestine of ruminant animals, finding an effective phage intervention has been challenging.^205^ Whereas oral phage dosage did not affect *E. coli* O157:H7 populations in sheep, it was effective against *E. coli* O157:H7 populations in mice.^214^ In general, phages can lower the levels of foodborne pathogens in animals and control slaughterhouse pathogen burden.^231^ Although the phage therapy is not a panacea for preventing all foodborne illnesses, it can be used in a multi-hurdle system to reduce enterohemorrhagic *E. coli* (EHEC) transmission from farm to fork.^214^

The excessive use of antibiotics can lead to the emergence of multidrug-resistant (MDR) pathogens in pets, resulting in financial and health issues,^232,233^ which may be prevented by phage therapy. For example, one dose of anti-K1 administered intramuscularly was more effective than multiple doses of conventional antibiotics (tetracycline, ampicillin, and chloramphenicol) in *E. coli-*infected calves.^234^ A recent study reported that 75% of veterinarians and pet owners chose phage treatment over antibiotics as a therapeutic method for companion animals. They proposed that phage treatment is used as an alternative to antibiotics as this treatment does not cause adverse effects.^235^ The treatment of animal infections by phage therapy has several advantages, including the phage’s ability to evolve, multiplication at the site of infection, and high specificity.^236^ Therefore, the phage therapy on animals may increase in the future. However, the use of phage treatment in animals may be limited due to:

- Requirement for high bacterial concentration, which is necessary for the phage to proliferate and lyse bacteria. If administered too rapidly, phages will inactivate due to absence of bacteria, thus requiring greater dosages later. In this regard, the phage treatment requires a high multiplicity of infection, which means a higher phage titer than the titer of the target bacteria,^237^
- Poor understanding of phage kinetics,^237^
- Presence of phage-resistant bacteria,^237^
- Specificity of phages, which precludes broad-spectrum application and often limits treatment scope. However, this characteristic is also considered advantageous.^139^

## 11. Phage-derived novel antimicrobial substances

Recent decades have seen an increasing trend in the prevalence of microorganisms that are MDR, extensively drug-resistant, and even pan-drug-resistant.^238^ The increase in drug resistance in bacteria has led to the emergence of new antibiotics and treatment strategies. As the phages multiply by utilizing the bacterial cellular machinery and subsequently destroy the cell, they can be used therapeutically as bacteriolytic agents. Phages can also play a significant role in the discovery of new antimicrobial substances. Phages produce bacteriolytic enzymes and polypeptides, which offer several advantages over the conventional antibiotics, particularly in biosafety and specificity. Most importantly, phages lyse the target bacteria without infecting normal or beneficial bacteria.^239^ Thus, living phages and vaccines employing phage genes can be used as therapeutic agents to address the global problem of increasing antibiotic resistance.^240^

### 11.1. Enzymes and peptides produced by phages as antimicrobial substances

Phages produce various enzymes, including lysin,^241^ endonuclease V,^242^ lysozyme,^243^ integrase,^244^ methylcarbamoylase,^245^ and DNA adenine methylase.^246^ Lysin, especially endolysin, can prevent biofilm formation or destroy the preformed biofilms.^238^ Endolysin can also hydrolyze the peptidoglycan layers of bacteria during cell lysis and release a progeny of virions, which subsequently destroy the bacterial host.^241^ Endolysin can therefore be used as an antibacterial agent to damage the cell walls of bacteria, leading to cell death via osmotic pressure. Absence of the outer membrane of Gram-positive bacteria allows endolysin to rapidly degrade the peptidoglycan layers.^241^ Thus, endolysin can be used to kill specific pathogenic bacteria in the gut without affecting the normal gut flora. For example, endolysin CD27L_EAD from Phage ΦCD27 is used as a therapeutic agent to treat *C. difficile*, which causes life-threatening diarrheal disease.^247^ However, in the case of gram-negative bacteria, the outer membrane with LPS inhibits the endolysin access to the peptidoglycan.^241^

Similarly, lysozymes derived from phages can hydrolyze the peptidoglycan layer of bacteria. For example, Gp105 from an *Enterobacter* phage is a lysozyme, murein hydrolase, which belongs to the glycoside hydrolase family 24. Murein hydrolases have strong antibacterial activity against gram-negative pathogens, such as *E. cloacae*, *K. pneumoniae*, and *P. aeruginosa*,^243^ which are associated with various gastrointestinal diseases or infections, and also affect the other gut microbes.^248–250^ Furthermore, endonucleases derived from phages can cleave the DNA molecules of the host bacteria. Ref, an endonuclease belonging to the HNH superfamily, can cleave DNA to which the RecA protein is bound^251^

There are also enzymes that can support the phage survival in the host bacteria, although these are not directly antibacterial. For example, methylcarbamoylase is responsible for methylcarbamoylation of adenine residues of DNA using acetyl CoA, which protects the viral DNA from bacterial restriction enzymes.^245^ Integrases, an enzyme group derived from phages, facilitate DNA recombination between the phage and the bacterial attachment site. The site-specific recombination of integrase can create genetic manipulations, which aid in the survival of phages in the host bacteria.^244^ Another enzyme, DNA adenine methylase (DAM), plays a significant role in the lifecycle regulation of phages. DAM plays a central role in the lysogenic and lytic states. DAM methylates the *rha* anti-repressor gene; once the methylation is removed, the homologous prophage repressor protein (CI) becomes repressed, which switches to the lytic cycle and subsequently destroys the host bacteria.^246^

Additionally, the phage genes produce several short peptides, such as terminase of the T4 phage, which plays a crucial role in the proper packaging of the phage genome into the capsid,^252^ maturation protein A of the *Escherichia* phage MS2, which assists in the assembly of the viral capsid during the maturation process,^253^ and the capsid protein of the *Enterobacteria* phage R17, which protects the viral genetic material and ensures a safe entry to the host cells during infection.^254^ The phage-derived peptides and their mode of action against target bacteria, such as *E. coli, P. aeruginosa*, and *S. aureus*, associated with gastrointestinal infection^250,255,256^ are summarized in the Table 3 below.

**Table 3.** Phage-derived antimicrobial peptides and mechanisms of action.

Author and year	Study type	Peptides	Targeted pathogen	Mechanism of action	Reference
Bernhardt et al., (2001)	In vitro and in vivo	ΦX174 lysis protein	*E. coli*	Blocks cell-wall synthesis by inhibiting MraY catalyzed step in the pathway.	257
Yano & Rothman-Denes, (2011)	In vitro	N4 Gp8		Blocks replication by inhibiting DNA polymerization by DNA polymerase III holoenzyme.	258
Kiro et al., (2013)	In vitro and in vivo	T7 Gp0.4		Interrupts cell division by inhibiting the *E. coli* filamenting temperature-sensitive mutant Z division protein.	259
Bae et al., (2013)	In vitro and in silico	T7 Gp2		Blocks transcription by inhibiting host RNAP.	260
Wagemans et al., (2015)	In vitro and in silico	LUZ24 Mip (Gp4)	*P. aeruginosa*	Blocks transcription by inhibiting MvaT protein.	261
Van Den Bossche et al., (2016)	In vitro and in vivo	ΦKZ Dip (Gp37)		Inhibits the RNA degradation machinery of the host by binding with two RNA-binding sites of the RNase E.	262
Van Den Bossche et al., (2014)	In vitro	14-1 Gp12		Blocks transcription by interacting with α subunit of RNA polymerase.	263
J. Liu et al., (2004)	In vitro	77 ORF104	*S. aureus*	Blocks replication by interacting with DnaI.	264

### 11.2. Endolysins as therapeutic agents in animal models

Endolysins have a long history, as they were first used in 1959 as recombinant proteins to lyse bacteria.^265^ However, they have not yet been authorized for use in humans as a therapeutic agent.^266^ Endolysins were initially used therapeutically to prevent bacterial infection in animal models.^241^ In a study by Briers et al., the gut colonization of *Caenorhabditis elegans* model was treated by LoGT-008 endolysin, where the endolysin LoGT-008 degraded the peptidoglycan layer of *P. aeruginosa*.^267^ In another study by Peng *et al*., Lysin-Human Defensin (LHD) was used to treat *C. difficile*-infected mice. The treatment increased the survival rate of the animals to 100%, whereas the survival rate of untreated mice was only 60%. In the treated mice, the loads of spores and toxins in the feces was reduced and the symptoms of diarrhea were mitigated.^268^ Yoong et al. also performed a study in a mouse model with peritonitis, where *B. anthracis* was treated with the PlyPH endolysin. The N-terminal half of the protein was used as the catalytic domain, and the C-terminal half was used for binding to a polysaccharide epitope that degrades bonds in the peptidoglycan layer, causing the lysis of cell wall and destruction of the cell.^269^

## 12. Conclusion and future perspectives

Although the role of phages in shaping the gut microbiota has a long research history since its initial discovery,^21^ phages were eclipsed by antibiotics that were more effective and easier to produce. However, the field of phage therapy has gained new attention after the emergence of antibiotic resistance. In this review, we gathered evidence on modulation of the gut microbiota by phages and emphasized their potential as therapeutic agents. Despite substantial data demonstrating the role of phages in gut ecology, several aspects remain unclear, such as the extent to which phages shape the gut microbial community. Based on our review, we hypothesize that phages are essential drivers of the dynamics of the gut environment, inducing subtle but important changes in the microbial population structures. We conclude that it is essential to fully understand the mechanisms underlying the interactions between the phages and the microbiota of humans and other animals to effectively employ phage therapy and other phage-based medical applications. Understanding the ability of therapeutic phages to both control pathogenic bacterial growth and modulate the functions of the target human or animal is needed.

We discussed the efficacy of phage therapy in treating bacterial infections in humans and in animals, including cattle and pets. Phages can be engineered to target specific bacterial strains, allowing development of specialized treatment regimens for animals infected with particular pathogens. There is a tripartite interplay between the phage, its bacterial host, and the animal host that influences both health and disease states. Phage therapy has currently gained increasing attention due to its potential ability to treat MDR infections. However, there are several issues and factors that should be considered for its proper implementation. Even though phage therapy is considered safe and effective in specific situations, more research, regulatory development, and standardization are needed before acceptance in the medical practice. Consequently, several multidisciplinary approaches are needed to identify the possible complications and opportunities associated with phage therapy.

Current studies on the use of phages in poultry and other animals have provided encouraging findings to promote the development and use of phage-based products, not only to prevent antibiotic overuse but also to ensure food safety. Phages could also be used to regulate the homeostasis of the gastrointestinal tract in livestock animals to improve their health. However, the effect of phages on animals can be favorable or unfavorable. It is essential to understand the phage-bacteria coevolutionary mechanisms, pharmacodynamics, phage-bacteria contact, and phage-animal interactions to fully comprehend the impact of phages on health and disease. Specifically, further research is needed on the effect of phage administration on the health of the human host. There are very few studies on modulation of gut metabolites by phages. The modulation of gut metabolites has further consequences on both the gut bacterial community and the health of the patient. In addition, the effect of phage therapy on patient immune responses warrants further examination.

Further identification of the stimuli that activate the prophage induction is another issue that should be addressed in further studies, particularly the stimuli from bacterial and fungal microbiota components. The function of phages in amyloid release warrants further attention, for example in studies utilizing animal models. Specifically, determining the precise role of *E. coli*-derived amyloid in development of T1D may lead to novel diagnostic and therapeutic approaches. Additionally, although phage-derived peptides have been hypothesized to act as antimicrobial agents in several studies, testing in animal models has been insufficient.

Another promising research approach is the in vivo testing of phage-based vaccines. Various complex diseases, such as IBD, obesity, and type 2 diabetes could be treated with effective phage-based therapeutics with the discovery and isolation of gut microbiota-modulating phages. Further in vivo testing of phage-based vaccines is needed to evaluate the efficacy of these approaches. If successful, phage-based therapeutics could be an effective alternative to antimicrobial therapies in the future.

## Abbreviations

AIM2: Absent in melanoma 2
APC: Antigen presenting cell
ATCC: American Type Culture Collection
BAM: Bacteriophage Adherence to Mucin
CDI: *Clostridioides difficile* infection
CFU: Colony-forming unit
CE: Competitive exclusion
cGAMP synthase: cyclic GMP-AMP synthase
CXCL-10: C-X-C Motif Chemokine Ligand 10
DAM: DNA adenine methylase
DNA: Deoxyribonucleic Acid
EHEC: Enterohemorrhagic *E. coli*
H-NS: Histone-like Nucleoid Structuring
IAPP: Islet amyloid polypeptide
IFN-γ: Interferon-gamma
IL: Interleukin
IL-1β: Interleukin-1 beta
IRF: Interferon regulatory factor
LVAD: left ventricular assist device
MDR: Multiple Drug Resistant
MHC II: Major histocompatibility complex class II
NF-kβ: Nuclear factor kappa B
PAMPs: Pathogen-associated molecular patterns
PFU: Plaque Forming Units
pKa: Acid Dissociation Constant at Logarithmic Scale
PRR: Pattern recognition receptor
RIG-1: Retinoic acid-inducible gene-I
STEC: Shiga toxin-encoding prophages of *E. coli*
TCR: T-cell receptor
TLRs: Toll-like receptors
Type I IFN: Type I interferon
T1D: Type 1 diabetes

## Data Availability

The authors confirm that the data supporting the findings of this study are available within this article. All the data will the found in the reference section in the form of DOI/URLs.
